# Depression and health-related quality of life in critical COVID-19 survivors

**DOI:** 10.1192/j.eurpsy.2022.956

**Published:** 2022-09-01

**Authors:** J. Silva, S. Martins, A.R. Ferreira, J. Fernandes, T. Vieira, L. Fontes, N. Reis, A. Braga, I. Coimbra, J.A. Paiva, L. Fernandes

**Affiliations:** 1 Faculty of Medicine University of Porto (FMUP), Fmup, Porto, Portugal; 2 Faculty of Medicine - University Porto, Department Of Clinical Neuroscience And Mental Health And Center for health technology and services research (cintesis), Porto, Portugal; 3 Centro Hospitalar Universitário São João (CHUSJ), Intensive Care Medicine Department, Porto, Portugal; 4 Faculty of Medicine - University Porto, Department Of Medicine, Porto, Portugal; 5 Psychiatry Service, Centro Hospitalar Universitário De São João, Porto, Portugal

**Keywords:** Covid-19, Quality-of-life, Depression, Critical illness

## Abstract

**Introduction:**

Long-term neuropsychiatric consequences of critical illness are well known. Therefore, it is expected that critical COVID-19 patients might also present several psychiatric symptoms such as depression, with inevitable negative effect on health-related quality of life (HRQoL), commonly used as an indicator of illness and treatment impact.

**Objectives:**

To identify depressive symptoms in critical COVID-19 survivors and to examine its association with HRQoL domains.

**Methods:**

This preliminary study involved critical COVID-19 patients admitted into the Intensive Care Medicine Department (ICMD) of a University Hospital, between October and December of 2020. Patients with an ICMD length of stay (LoS)≤24h, terminal illness, major auditory loss, or inability to communicate at the follow-up time were excluded. From 1-2 months after discharge, all participants were evaluated by telephone at follow-up appointment, with Patient Health Questionnaire (PHQ-9) (depression) and EuroQol 5-dimension 5-level EQ-5D-5L (HRQoL). This study is part of the longitudinal MAPA project.

**Results:**

Eighty-three patients were included with a median age of 63 years (range: 31-86) and the majority were male (63%). The most reported problems on EQ-5D-5L domains were usual activities (82%) and mobility (76%). About 27% presented depressive symptoms, and with more problems of self-care (68%vs41%; p=0.029), pain/discomfort (86%vs49%; p=0.002), and anxiety/depression (96%vs54%; p<0.001).

**Conclusions:**

These preliminary results are in line in previous studies in critical COVID-19 survivors, with depression being associated with worse HRQoL. Bearing this in mind, follow-up approaches with an early screening and treatment of these psychiatric symptoms will be fundamental to optimize the recovery of these patients.

**Disclosure:**

No significant relationships.

